# Beyond Plasma: Blood Fraction-Specific Patterns of Magnesium, Calcium and Iron in Type 2 Diabetes Across Preserved and Reduced Estimated Glomerular Filtration Rate

**DOI:** 10.3390/jcm15218727

**Published:** 2026-11-09

**Authors:** Iwona Majak, Rafał Frankowski, Marcin Kosmalski, Joanna Leszczyńska, Marcin Basiak, Tadeusz Pietras, Monika Różycka-Kosmalska

**Affiliations:** 1 Institute of Food Technology and Analysis, Department of Biotechnology and Food Sciences, Lodz University of Technology, Lodz 90-924, Poland; iwona.majak@p.lodz.pl; 2 Department of Clinical Pharmacology, Medical University of Lodz, Lodz 90-153, Poland; rafal.frankowski@student.umed.lodz.pl (R.F.); marcin.kosmalski@umed.lodz.pl (M.K.); tadeusz.pietras@umed.lodz.pl (T.P.); 3 Institute of Food Technology and Analysis, Faculty of Biotechnology and Food Sciences, Lodz University of Technology, Lodz 90-924, Poland; joanna.leszczynska@p.lodz.pl; 4 Department of Internal Medicine and Clinical Pharmacology in Katowice, Medical University of Silesia in Katowice, Katowice 40-752, Poland; mbasiak@sum.edu.pl; 5 Department of Electrocardiology, Medical University of Lodz, Lodz 92-213, Poland

**Keywords:** diabetes mellitus type 2, renal dysfunction, diet, mineral elements

## Abstract

**Background:** Type 2 diabetes mellitus (T2DM) is associated with disturbances in mineral element homeostasis, yet data on calcium (Ca), magnesium (Mg) and iron (Fe) across multiple blood compartments in T2DM throughout the spectrum of renal function remain limited. **Objective:** To assess Ca, Mg and Fe concentrations in plasma, whole blood and erythrocytes of T2DM patients with reduced or preserved estimated glomerular filtration rate (eGFR), integrating three-day dietary records to explore, rather than definitively separate, metabolic and dietary drivers of imbalance. **Methods:** In this single-center, cross-sectional study, 91 hospitalized adults were divided into three groups: controls (*n* = 32), T2DM with preserved eGFR (≥60 mL/min/1.73 m^2^; n = 29), and T2DM with reduced eGFR (<60 mL/min/1.73 m^2^; n = 30). Renal function was assessed using eGFR calculated with the Modification of Diet in Renal Disease (MDRD) equation. The eGFR-based grouping did not constitute a diagnosis or staging of chronic kidney disease. Mg, Ca, and Fe concentrations were measured in plasma and whole blood using atomic absorption spectrometry. Plasma Ca concentrations were corrected for albumin, whereas blood cell Fe and Mg concentrations were calculated using individual hematocrit values. Blood cell Ca could not be quantified. Dietary intake was assessed from three-day food diaries completed during hospitalization (hospital diet). Between-group differences in mineral concentrations were assessed using Welch’s ANOVA followed by Games–Howell post hoc comparisons. Associations between calculated blood cell Fe concentrations and hematological indices were evaluated using Spearman’s rank correlation coefficient, with Benjamini–Hochberg correction for multiple comparisons. **Results:** Plasma Mg (*p* < 0.01) and albumin-corrected plasma Ca (*p* < 0.05) were lower in the T2DM group with eGFR < 60 mL/min/1.73 m^2^ than in controls; albumin-corrected plasma Ca was also lower in this group than in the T2DM group with eGFR ≥ 60 mL/min/1.73 m^2^ (*p* < 0.01); whole-blood Mg was higher in the T2DM group with eGFR ≥ 60 mL/min/1.73 m^2^ than in controls (*p* < 0.05). Whole-blood Fe was significantly higher in both T2DM groups than in controls (*p* < 0.05), whereas calculated blood cell Fe did not differ significantly between groups after hematocrit-based recalculation. Blood cell Mg did not differ significantly between groups. Dietary Ca intake was lower (*p* < 0.01) and Mg intake higher (*p* < 0.01) in T2DM than in controls. **Conclusions:** Mineral concentration differences in T2DM varied across blood compartments. Although whole-blood Fe was elevated in both T2DM groups, no significant differences were detected in hematocrit-adjusted blood cell Fe concentrations. These findings do not establish intracellular Fe accumulation or hepcidin-mediated sequestration. Further studies incorporating direct intracellular measurements, comprehensive Fe-status markers, and adjustment for clinical confounders are needed to determine the potential diagnostic value of mineral measurements beyond plasma.

## 1. Introduction

Type 2 diabetes mellitus (T2DM) leads to disturbances in the maintenance of selected minerals, such as iron (Fe), calcium (Ca) and magnesium (Mg) [1,2,3]. These disturbances contribute to dysfunction and complications affecting the eyesight, nerves, kidneys and large blood vessels [4], and mineral deficiencies may further exacerbate these risks [5,6]. The foundation of T2DM therapy remains a well-balanced diet and physical activity that reduce body weight and maintain it at a proper level [7]. In addition to dietary regulation, two contemporary endocrine axes dominate systemic handling of these minerals in T2DM with renal comorbidity and frame the present study. Hepcidin, a 25-amino-acid hepatic peptide, binds the only known cellular Fe exporter ferroportin, triggering its internalization and degradation [8,9,10]. Inflammatory cytokines, particularly interleukin-6, and the iron-loaded state of macrophages drive hepcidin expression; the resulting suppression of ferroportin traps Fe inside enterocytes, macrophages and erythroid-lineage cells, producing the hypoferremia and intracellular Fe sequestration that characterize anemia of inflammation [8,10]. This axis is dysregulated in T2DM, where chronic low-grade inflammation sustains hepcidin elevation [9,11]; increased hepcidin expression has been linked to intracellular Fe sequestration and diabetic complications, while underexpression contributes to body Fe overload and oxidative β-cell injury [9]. In chronic kidney disease (CKD), fibroblast growth factor 23 rises early, α-Klotho expression falls and 1α-hydroxylase activity declines, driving secondary hyperparathyroidism, hypocalcemia, hyperphosphatemia, bone disease and vascular calcification, collectively termed CKD mineral and bone disorder [12]. Decreased renal α-Klotho expression has been documented in early diabetic nephropathy, with consequences for urinary Ca excretion [13]. These mechanisms explain why the plasma Ca/Mg ratio has been proposed as a candidate integrative readout of mineral status in T2DM.

Dietary mineral element supplementation is not recommended when concentrations are normal; it is common in daily clinical practice to encounter patients whose diets either lack adequate nutrients or provide them in inadequate proportions. Diets should be balanced and adjusted according to age, body weight and eating habits [14], and modified when kidney dysfunction is present [15]. This raises the question of whether reduced renal function in patients with T2DM is associated with differences in mineral status and dietary mineral intake, and whether measurements across different blood fractions may provide information beyond plasma-based assessments [16]. Sodium (Na) intake is a determinant of Ca and Mg handling rather than a neutral background: in human balance studies, low Na intake was associated with negative Ca and Mg balances [17] and pooled balance data indicate that Na intake correlates with the balances of these elements once extreme intakes are excluded [18]. Because all participants in this study received a hospital-prescribed diet whose salt content need not equal that of their habitual home diet, Na intake was quantified from the same three-day food diaries used for the mineral elements and is reported alongside them; it is treated as a candidate confounder of the Ca and Mg comparisons rather than as a controlled variable.

The main objectives of this study were to assess Ca, Mg and Fe concentrations in plasma, whole blood and erythrocytes of T2DM patients with preserved or reduced estimated glomerular filtration rate (eGFR), rather than formally established CKD, since eGFR was determined at a single time point without albuminuria assessment, and to compare the results with a control group free of carbohydrate metabolism disorders and with preserved renal function.

## 2. Materials and Methods

### 2.1. Study Design and Participants

This was a single-centre, cross-sectional study conducted in the Department of Internal Medicine, Diabetology and Clinical Pharmacology of the Regional Specialist Hospital in Zgierz, Poland, between 2010 and 2012. All participants were patients hospitalised in this department during the recruitment period. Ninety-one patients (aged 41–89 years; 68.05 ± 12.70 years; 36 men, 55 women) were enrolled: controls (16 women, 16 men, n = 32), T2DM with eGFR ≥ 60 mL/min/1.73 m^2^ (17 women, 12 men, n = 29) and T2DM with eGFR < 60 mL/min/1.73 m^2^ (22 women, 8 men, n = 30). The control group was recruited from the same department and during the same period as the two T2DM groups, so that all three groups were hospitalised patients in a single ward and all received the same standard hospital diet prepared by the hospital kitchen. Written informed consent was obtained from every participant. Inclusion criteria: (i) age ≥ 18 years; (ii) hospitalisation in the study department during the recruitment period; (iii) for the T2DM groups: a history of T2DM or newly diagnosed T2DM meeting the diagnostic criteria of the Polish Diabetes Association (fasting plasma glucose ≥ 126 mg/dL confirmed on a second measurement, 2-h plasma glucose ≥ 200 mg/dL in a 75-g oral glucose tolerance test or random plasma glucose ≥ 200 mg/dL with typical symptoms); (iv) for the control group: no history of diabetes, normal fasting glucose and HbA1c, and eGFR ≥ 60 mL/min/1.73 m^2^; (v) completion of the 3-day food diary and provision of the full set of blood samples for mineral determination.

Exclusion criteria, applied identically to the control group and to both T2DM groups and defined before data analysis: (1) age < 18 years; (2) pregnancy or lactation; (3) use of supplements containing mineral elements within 4 weeks before admission, because such supplements directly alter the exposure of interest and cannot be reliably quantified from food diaries; (4) treatment with diuretics (thiazide, thiazide-like, loop or potassium-sparing agents) or with medicinal products or supplements that substantially alter the intestinal microbiota or the absorption of nutrients, e.g., proton pump inhibitors, histamine H_2_-receptor antagonists, systemic antibiotics within 4 weeks before admission, laxatives and orlistat; (5) macroangiopathic complications of T2DM, defined as documented atherosclerotic cardiovascular disease: previous myocardial infarction, coronary revascularisation (percutaneous coronary intervention or coronary artery bypass grafting) or angiographically confirmed coronary stenosis; ischaemic stroke or transient ischaemic attack confirmed by imaging or by neurological documentation; and peripheral arterial disease (intermittent claudication with imaging-confirmed arterial stenosis or occlusion, previous peripheral revascularisation, or amputation for ischaemic causes). The diagnosis had to be documented in the hospital records, i.e., supported by a discharge diagnosis, an imaging or angiography report, or specific pharmacological treatment of established atherosclerotic disease. This criterion restricts the T2DM groups to patients without clinically overt macrovascular disease and therefore to a vascularly healthier subset than the typical patient in an internal medicine and diabetology ward; (6) acute kidney injury or unstable renal function, as defined below; (7) acute infection or active inflammatory disease; (8) active malignancy, because malignancy affects plasma ferritin to such an extent that it cannot be used to assess iron status, and because altered liver function and inflammation can falsely elevate markers of iron stores; (9) other conditions precluding the measurement or interpretation of the analysed parameters: (a) renal replacement therapy (haemodialysis, peritoneal dialysis) or kidney transplantation, because creatinine-based estimates of eGFR are not valid in these patients; (b) chronic liver disease with impaired synthetic function or cholestasis, because hepatic reticuloendothelial clearance of ferritin is impaired in liver dysfunction, which raises plasma ferritin independently of iron status; (c) history of malignancy within the previous 5 years; (d) haemoglobinopathies (thalassaemia, sickle cell disease); (e) blood transfusion within 3 months before admission; (f) oral or intravenous iron therapy, or vitamin D supplementation, within 3 months before admission; (g) alcohol abuse, because excessive alcohol consumption increases magnesium losses; (h) inability to provide informed consent or to complete the 3-day food diary.

The primary indication for admission was recorded for each participant at enrolment. Controls were admitted to the hospital predominantly for elective diagnostic evaluation of arterial hypertension and metabolic syndrome and for cardiac causes, while patients with T2DM were admitted predominantly for decompensated glycaemia, arterial hypertension requiring treatment adjustment and elective diagnostic evaluation. The majority of participants in all three groups were admitted electively for diagnostic evaluation or treatment adjustment rather than as emergency admissions.

Renal function was assessed using the estimated glomerular filtration rate (eGFR), calculated from creatinine concentrations using the Modification of Diet in Renal Disease (MDRD) equation [19,20,21,22,23,24]. For the purposes of statistical analysis, patients with T2DM were divided into two groups: preserved eGFR (≥60 mL/min/1.73 m^2^) and reduced eGFR (<60 mL/min/1.73 m^2^). This classification was based exclusively on the eGFR threshold, without further subdivision into individual GFR categories.

Chronic kidney disease (CKD) could not be formally established because the persistence of reduced kidney function for at least three months was not verified and urinary albumin excretion was not assessed. Accordingly, the terms “preserved eGFR” and “reduced eGFR” are used throughout the manuscript to describe renal function rather than confirmed CKD status.

The MDRD equation was used because it was routinely employed in the study laboratory during the data collection period (2010–2012). The potential implications of using MDRD rather than the currently recommended Chronic Kidney Disease Epidemiology Collaboration (CKD-EPI) equation are acknowledged as a methodological limitation. To assess the potential impact of the eGFR estimation equation on patient classification, an exploratory sensitivity analysis was performed using the 2021 CKD-EPI creatinine equation. Individual creatinine concentrations, age, and sex were available for all 91 participants. However, the analytical methods and isotope dilution mass spectrometry (IDMS) traceability of the historical creatinine measurements could not be verified. Therefore, CKD-EPI estimates were considered exploratory, and the original eGFR-based classification was retained for the primary analyses.

Stability of renal function. Patients were classified as having stable renal function only when no acute kidney injury was documented in the hospital records and plasma creatinine on admission and on the day of blood sampling differed by less than 0.3 mg/dL (26.5 µmol/L). Acute kidney injury is defined as an increase in serum creatinine of ≥0.3 mg/dL within 48 h, or an increase to ≥1.5 times baseline, known or presumed to have occurred within the preceding 7 days, and/or urine output < 0.5 mL/kg/h for 6 h [25,26]; establishing this diagnosis requires at least two creatinine values within the relevant time window [27], and the baseline creatinine is frequently unknown in patients admitted to the hospital [28]. Serum creatinine is an imperfect marker of eGFR and may change for non-renal reasons, and kidney injury may be present with no change in creatinine at all [29]; in patients with unstable renal function, creatinine-based estimates are not reliable [30], and serum creatinine may remain within the reference range in the early phase of acute kidney injury [31]. Because renal function was assessed at a single time point, the urinary albumin-to-creatinine ratio was not quantified, and a pre-admission baseline creatinine was frequently unavailable, chronicity could not be verified and stability could be assessed only within the hospitalisation and not relative to the patients’ baseline values; the strata are therefore GFR-based (Section 2.1). We did not measure urinary creatinine clearance over a defined collection period because we did not collect urine in this study.

### 2.2. Laboratory Analyses and Erythrocyte Calculation

Blood sampling for mineral determinations was performed on day 3 of the 5-day hospital stay, in the morning after an overnight fast (≥ 8 h), before breakfast. In principle, samples drawn early in the stay would reflect pre-admission (habitual) status, whereas later sampling would reflect the hospital environment; day 3 was chosen as a compromise that places the sample within the 3-day dietary record (days 2–4), so that plasma concentrations and the documented dietary intake refer to the same observation window, thereby limiting hospital exposure to approximately 48 h. This choice is methodologically defensible for Ca and Mg in particular: plasma Ca and Mg concentrations are tightly homeostatically regulated and do not respond measurably to short-term changes in dietary intake [32,33]; exposure to the hospital diet for approximately 48 h therefore does not materially confound these plasma measurements relative to pre-admission status. Plasma Fe is more labile; several authors report circadian variation [34,35], although not all studies confirm a consistent within-day pattern [36]. Fasting morning sampling was used to standardize its determination.

Because the blood cell fraction reflects the mineral content of cells with an approximately 120-day lifespan, the calculated blood cell values are less sensitive to short-term fluctuation, including the day of sampling, than plasma concentrations; being derived from whole-blood and plasma measurements and from the individual hematocrit, however, they inherit the variability of both input measurements and of the hematocrit itself.

Albumin correction of plasma calcium. Total calcium concentrations were measured in plasma using atomic absorption spectrometry and subsequently corrected for albumin concentration according to the following equation: corrected Ca (mg/dL) = measured total Ca (mg/dL) + 0.8 × (4 − albumin [g/dL]) [37]. The albumin-corrected values were converted to mg/L and are reported in Table 1. This correction was applied to plasma calcium concentrations only; whole-blood calcium concentrations are presented without albumin correction.

Hypoalbuminemia is common in T2DM with renal dysfunction and uncorrected total Ca can falsely suggest hypocalcemia; albumin-corrected Ca avoids this confounding [38]. Mg, Ca, and Fe concentrations were determined in whole blood and the separated plasma fraction using atomic absorption spectrometry (AAS), as described in the Supplementary Materials (Tables S1–S3). Blood cell concentrations of Mg and Fe were not measured directly but were calculated separately for each participant using the corresponding whole-blood and plasma concentrations and the individual hematocrit value, according to the following equation:

C_cells = [C_WB − (1 − Hct) × C_plasma]/Hct

where C_cells represents the calculated mineral concentration in the blood cell fraction (mg/L), C_WB is the measured whole-blood concentration (mg/L), C_plasma is the measured plasma concentration (mg/L), and Hct is the hematocrit expressed as a fraction (0–1).

This calculation assumes that the hematocrit represents the volume fraction occupied by blood cells and that the remaining blood volume corresponds to plasma. Because blood cell concentrations were derived indirectly from two analytical measurements and hematocrit, their uncertainty reflects the combined variability of these parameters. Direct intracellular measurement was not available. Erythrocyte Ca could not be quantified owing to insufficient methodological sensitivity in the diluted lysate and was recorded as “Not Determined” in Table 1.

### 2.3. Dietary Assessment

All patients followed dietary recommendations for the Polish population issued by the National Institute of Public Health [39] during a 5-day hospital stay and kept 3-day food diaries (days 2–4, including one weekend day), collected and reviewed daily by a trained dietitian. The diaries were used to quantify the intake of five elements: Na, K, Ca, Mg and Fe—reported in Table 2 and Section 3.5. Na was quantified from these same diaries rather than assessed independently of the diet: no 24-h urine collection was performed, so no urinary Na, Ca, Mg or Fe excretion data are available; the Na content of the hospital menu as prepared was not available to us, and pre-admission (habitual) Na intake was not recorded. Because the diaries were completed during hospitalization, the recorded sodium values describe the hospital diet as consumed and cannot be compared with the habitual home diet. Na was treated as a candidate confounder of the Ca and Mg comparisons rather than as a controlled variable. No energy-based (nutrient-density) adjustments were applied; intakes were expressed as absolute daily amounts. All patients refrained from additional physical activity during the 5-day period. Accordingly, the dietary analyses cannot, by themselves, separate metabolic from habitual dietary drivers, and no inference about habitual intake is made. The use of dietary supplements containing mineral elements was an exclusion criterion; detailed information on all medications and supplements was collected from each patient.

### 2.4. Anthropometrics and Clinical Variables

Blood pressure (BP) and anthropometrics (weight, height, waist and hip circumferences) were measured by trained study personnel, rather than self-reported. Body weight and height were measured with a calibrated column scale with an integrated stadiometer (Seca 704; CE-marked, Directive 93/42/EEC), with participants in light clothing and without shoes, and body mass index was calculated. Waist circumference was measured with a flexible, non-extensible tape (precision 0.1 cm) at the midpoint between the lower border of the last palpable rib and the iliac crest, at the end of normal expiration, and hip circumference was measured at the widest portion of the buttocks, following the WHO STEPS protocol [40] (among the protocols recommended by the IAS/ICCR consensus [41]); each circumference was measured twice and the mean of the two measurements was used, and waist–hip ratio (WHR) was calculated. Standard laboratory tests covered renal function (creatinine, urea), carbohydrate metabolism (fasting glucose (FG), glycated hemoglobin (HbA1c)), lipid metabolism (total cholesterol (TCH), low-density lipoprotein (LDL), high-density lipoprotein (HDL), triglycerides (TG)), liver function (activity of alanine aminotransferase (ALT), aspartate aminotransferase (AST), gamma-glutamyl transpeptidase (GGTP) and total bilirubin concentrations), uric acid, potassium (K), sodium (Na) and basic morphology.

### 2.5. Statistical Analysis

Data are presented as means ± standard deviations (SD). Differences in mineral concentrations among the three study groups (controls, T2DM with eGFR ≥ 60 mL/min/1.73 m^2^, and T2DM with eGFR < 60 mL/min/1.73 m^2^) were assessed using Welch’s one-way analysis of variance (ANOVA), which accommodates unequal group sizes and heterogeneity of variances. Pairwise comparisons were performed using the Games–Howell post hoc test, which accounts for unequal variances and multiple comparisons.

An exploratory sensitivity analysis was conducted to assess the potential impact of the eGFR estimation equation on patient classification and selected clinical outcomes. eGFR was recalculated for all 91 participants using the 2021 CKD-EPI creatinine equation, based on individual creatinine concentrations, age, and sex, under the assumption of IDMS-standardized creatinine measurements. Classification according to the threshold of 60 mL/min/1.73 m^2^ was compared with the original eGFR classification. Selected clinical and hematological parameters were subsequently reassessed using Welch’s ANOVA following the exploratory CKD-EPI-based grouping. A participant from the control group whose recalculated eGFR fell below 60 mL/min/1.73 m^2^ was not included in the three-group comparison.

The threshold for statistical significance was set at *p* < 0.05. The primary between-group comparisons of mineral concentrations were evaluated without an additional correction across different elements and blood fractions. Therefore, these results should be interpreted with consideration of the increased risk of Type I error associated with multiple testing.

In an additional exploratory analysis, associations between calculated blood cell iron concentrations and hematological indices (MCV, MCH, and MCHC) were assessed using Spearman’s rank correlation coefficient. The Benjamini–Hochberg procedure was applied to control the false discovery rate across nine within-group correlation tests. Two-sided *p*-values were reported. The original statistical analyses were conducted using the complete study cohort of 91 participants and the original dataset, which is no longer accessible for retrospective reanalysis. During manuscript revision, additional exploratory correlation analyses were performed using individual-level records recovered from archived documentation. Complete matched data required for these additional analyses were available for 45 participants (18 controls, 17 patients with T2DM and preserved eGFR, and 10 patients with T2DM and reduced eGFR). These supplementary analyses followed a complete-case approach, without imputation of missing observations. Spearman’s rank correlation coefficients were calculated separately for each group, and the Benjamini–Hochberg procedure was applied across the nine within-group correlations involving calculated blood cell Fe concentrations and MCV, MCH, and MCHC.

Differences in anthropometric, biochemical, hematological, and dietary parameters between independent study groups were assessed using the nonparametric Mann–Whitney U test. This method was selected to accommodate departures from normality in the distributions of the analyzed variables. Comparisons were performed between the control group and the respective T2DM groups, as well as between the two T2DM groups stratified by renal function, where applicable. Dietary intake data were analyzed separately by comparing the control group with the combined T2DM cohort, reflecting the structure of the available dietary dataset.

Statistical analyses were performed using Jamovi 2.7.31 and Principal Component Analysis Calculator. Supplementary Figures S1–S3 present within-group covariance; Figure S4 presents inter-group data; within groups, each variable appeared independent, justifying the adopted division, while clusters of variables appeared between groups.

## 3. Results

### 3.1. Clinical Characteristics of Study Participants

The demographic and clinical characteristics of all participants are shown in Supplementary Table S4. The three groups differed in age and sex distribution, with a particularly high proportion of women in the reduced-GFR T2DM group (22 of 30); the potential confounding effect of these imbalances is addressed in Section 4.3 and Section 4.6. Most patients had diabetes for several years (median 6.69 (3.25–10) years) and were treated with insulin (n = 21), oral agents (n = 25), or both (n = 11); the cohort was predominantly female. Sixty-seven participants were overweight, including 35 with obesity (BMI ≥ 30 kg/m^2^).

Exploratory within-group comparisons did not reveal significant sex differences for the mineral outcomes, but these analyses were underpowered; the sex imbalance is discussed in Section 4.3 and Section 4.6. Both T2DM groups differed significantly from controls in BMI, blood glucose and HbA1c; the eGFR ≥ 60 group had significantly higher AST and ALT, and the eGFR < 60 group had higher uric acid, urea and creatinine. Between the two T2DM groups, whole-blood K, uric acid, urea and creatinine differed significantly. These details are provided in Supplementary Tables S5–S7.

### 3.2. Results of Magnesium Determination in Whole Blood, Plasma, and Blood Cells

Table 1 and Supplementary Figure S5 show that plasma Mg concentrations were significantly lower in the T2DM group with eGFR < 60 mL/min/1.73 m^2^ than in controls (*p* < 0.01), whereas the difference in the T2DM group with preserved eGFR was not statistically significant. Whole-blood Mg was significantly higher in the T2DM group with eGFR ≥ 60 mL/min/1.73 m^2^ than in controls (*p* < 0.05) and differed significantly from the T2DM group with reduced eGFR (*p* < 0.01). No statistically significant between-group differences were observed in calculated blood cell Mg concentrations. Plasma Mg concentrations expressed in mmol/L (1 mmol/L = 24.3 mg/L) are provided in the Supplementary Materials.

### 3.3. Results of Calcium Determination in Whole Blood and Plasma

Table 1 and Supplementary Figure S6 show that albumin-corrected plasma Ca differed significantly between the two T2DM groups: it was lower in the T2DM group with eGFR < 60 mL/min/1.73 m^2^ than in the T2DM group with eGFR ≥ 60 mL/min/1.73 m^2^ (80.9 ± 4.8 vs. 89.0 ± 8.7 mg/L; *p* < 0.01) and was also significantly lower than in controls (*p* < 0.05). In the T2DM group with preserved eGFR, plasma Ca was numerically higher than in controls (89.0 ± 8.7 vs. 86.7 ± 11.3 mg/L), but this difference was not statistically significant. Whole-blood Ca did not differ between groups.

### 3.4. Results of Iron Determination in Whole Blood, Plasma, and Blood Cells

Table 1 and Supplementary Figure S7 show that whole-blood Fe concentrations were significantly higher in both T2DM groups than in controls (*p* < 0.05). Plasma Fe was significantly higher in patients with T2DM and eGFR < 60 mL/min/1.73 m^2^ than in controls (*p* < 0.05), whereas the difference between the two T2DM groups was not statistically significant. Following hematocrit-based recalculation, blood cell Fe concentrations were numerically higher in both T2DM groups than in controls; however, these differences did not reach statistical significance.

### 3.5. Dietary Intake of Mineral Elements

Table 2 reports the 3-day food diary intake of Na, K, Ca, Mg, and Fe (control, n = 32; aggregated T2DM, n = 59; Mann–Whitney U test on the control vs. aggregated T2DM contrast). Mean Na intake was lower in T2DM (2328.52 ± 434.51 vs. 2948.01 ± 364.21 mg/day; *p* < 0.01); K intake was higher (3033.39 ± 585.76 vs. 2358.83 ± 535.98 mg/day; *p* < 0.01); Ca intake was approximately 40% lower (438.28 ± 225.43 vs. 730.72 ± 431.22 mg/day; *p* < 0.01); Mg intake was modestly higher (309.36 ± 44.65 vs. 249.63 ± 53.21 mg/day; *p* < 0.01); Fe did not differ (13.24 ± 7.20 vs. 11.76 ± 6.10 mg/day; *p* > 0.05). The lower Na and Ca and higher Mg intakes in T2DM are consistent with the dietary counselling routinely delivered in this cohort; because the diaries were completed in hospital, these values reflect the hospital-prescribed diet. Disaggregated per-stratum intakes are recorded as “ND” because the original tabulation aggregated T2DM patients into a single cohort (see limitation, Section 4.6).

### 3.6. Correlations Between Calculated Blood Cell Iron Concentrations and Hematological Indices

Hematological parameters and mineral concentrations were measured in the full study cohort, but the individual-level records required for the additional correlation analyses could be recovered from the archived documentation for only 45 of the 91 participants: 18 controls, 17 patients with T2DM and preserved eGFR (≥60 mL/min/1.73 m^2^) and 10 patients with T2DM and reduced eGFR (<60 mL/min/1.73 m^2^). Recovery was therefore unequal across groups (56.3%, 58.6% and 33.3% of the respective groups), so the analysed subset cannot be assumed to be representative of the original cohort. Correlations between calculated blood cell Fe concentrations and the hematological indices that do not enter that calculation were therefore evaluated exclusively in this subset, using a complete-case approach without imputation; the analysis is exploratory and underpowered, particularly for the reduced-eGFR subgroup (n = 10).

The red-cell indices available for the full cohort (n = 91; Supplementary Table S7) were then examined as potential explanations for the numerical between-group differences in calculated blood cell Fe. Haemoglobin, haematocrit, MCV and MCH did not differ in any contrast (all *p* > 0.05), whereas MCHC differed between groups (Welch’s ANOVA, *p* = 0.0068). The numerical differences in calculated blood cell Fe were therefore not accompanied by a parallel shift in erythroid mass or in red-cell size, which does not support differences in these indices as an explanation for the observed differences.

Spearman’s rank correlation analysis revealed nominally significant associations between calculated blood cell Fe concentrations and mean corpuscular hemoglobin concentration in the T2DM groups, with a positive correlation in participants with eGFR ≥ 60 mL/min/1.73 m^2^ (r_s_ = 0.584, *p* = 0.0138) and a negative correlation in those with eGFR < 60 mL/min/1.73 m^2^ (r_s_ = −0.733, *p* = 0.0158). However, neither association remained statistically significant after correction for multiple comparisons using the Benjamini–Hochberg procedure (adjusted *p* = 0.0711 for both).

No significant correlations with mean corpuscular volume or mean corpuscular hemoglobin were identified. Correlations with hemoglobin and hematocrit were not formally tested: calculated blood cell Fe is derived from hematocrit by definition, and the majority of whole-blood Fe is incorporated into hemoglobin, so an association with either parameter would arise mathematically and would not be interpretable as an independent biological relationship. The correlation analysis was therefore restricted to the hematological indices that are not used in the calculation of blood cell Fe. Detailed correlation coefficients and corresponding *p*-values are presented in Supplementary Table S8.

### 3.7. Sensitivity Analysis of eGFR Classification

In an exploratory sensitivity analysis, eGFR was recalculated for all 91 participants using the 2021 CKD-EPI creatinine equation. Compared with the original eGFR classification, five participants crossed the threshold of 60 mL/min/1.73 m^2^. Four patients with T2DM were reclassified from reduced to preserved eGFR, while one control participant had a recalculated eGFR below 60 mL/min/1.73 m^2^.

Reassessment of selected clinical and hematological parameters using the exploratory CKD-EPI-based grouping showed broadly similar patterns of statistical significance. Between-group differences in BMI and mean corpuscular hemoglobin concentration (MCHC) remained statistically significant, although the significance of MCHC was less pronounced (Welch’s ANOVA: *p* = 0.0068 with the original grouping versus *p* = 0.0487 with CKD-EPI-based grouping). Other evaluated parameters remained statistically non-significant under both classifications.

These analyses were exploratory because the standardization of the historical creatinine measurements could not be verified.

## 4. Discussion

### 4.1. Magnesium

Our study identified compartment-dependent differences in Mg concentrations among the study groups. Plasma Mg was significantly lower in patients with T2DM and eGFR < 60 mL/min/1.73 m^2^ than in controls, whereas the reduction observed in the T2DM group with preserved eGFR did not reach statistical significance. Previous studies have associated lower plasma Mg concentrations with impaired metabolic control in T2DM [42,43,44,45]. In whole blood, Mg was significantly higher in the preserved-eGFR T2DM group than in controls (38.0 vs. 35.0 mg/L, *p* < 0.05), whereas hematocrit-adjusted blood cell Mg concentrations did not differ significantly among the groups.

An important observation is that the differences detected in circulating Mg concentrations were not accompanied by significant differences in the calculated blood cell fraction. Although Mg intake recorded during hospitalization was higher in patients with T2DM, these dietary data cannot establish a relationship between intake and mineral distribution. Altered absorption, renal losses, and changes in Mg compartmentalization remain possible explanations, but none was directly assessed in the present study. One further confounder merits explicit attention because it differs systematically between the hospital and the home setting: sodium status is a determinant of Mg handling rather than a neutral background. In human balance studies, low Na intake was associated with negative Ca and Mg balances [17], and pooled balance data indicate that Na intake correlates with the balances of these elements once extreme intakes are excluded [18]. In our cohort, Na intake quantified from the same three-day diaries differed significantly between T2DM patients and controls (2328.52 ± 434.51 mg/day in T2DM vs. 2948.01 ± 364.21 mg/day in controls; *p* < 0.01), whereas the salt content of a hospital-prescribed diet need not match that of the habitual home diet. A sodium-driven shift in Mg balance—and, to a lesser extent, in Ca balance, which is more tightly homeostatically controlled—between the home and hospital settings therefore cannot be excluded as a contributor to the between-group differences in plasma Mg. Sodium is thus acknowledged as an unresolved confounder rather than a controlled variable, and the Mg findings should be read with this caveat.

The plasma Ca/Mg ratio may outperform single markers: in 891 T2DM patients it discriminated poor glycemic control with area under curve (AUC) 0.672 vs. Mg 0.650, K 0.611 and Ca 0.578 [46]; in diabetic coronary artery disease, a cutoff of 2.55 was identified, with CAD prevalence rising from 18.5% below to 46.9% above it [47]; a proposed unified plasma Mg/Ca scale predicted mortality and 5-year outcomes better than Mg alone [48].

Total plasma Mg is an imperfect proxy for body Mg status: intracellular depletion can coexist with normal total Mg, particularly in older and diabetic subjects [49]. In a prospective T2DM cohort of 329 adults, hypomagnesemia prevalence ranged from 10.0% (ionized Mg) to 56.2% (total Mg), and the iMg/tMg ratio was associated with HbA1c and retinopathy [50]. Ionized Mg is increasingly advocated as the operational marker of choice in T2DM [51]. We did not calculate the Ca/Mg ratio or measure iMg because total Mg is the routine hospital assay; ionized Mg requires point-of-care ion-selective instruments that were unavailable; and retrospective ratio computation on already-reported means would conflate unit heterogeneity. An internally validated ratio scale in identical units is required [48]. Both metrics are priorities for follow-up; the present total-Mg-based observations are a necessary but partial readout, and dietary or supplemental Mg recommendations should remain preliminary pending intervention studies including absorption and status measures. Finally, because the groups differed in age, sex, BMI and glycemic control and the analyses were univariate, residual confounding of these Mg differences cannot be excluded (Section 4.6).

### 4.2. Calcium

Plasma Ca was significantly lower in patients with T2DM and impaired renal function than in controls. Authors have reported elevated plasma Ca in diabetes [42] and linked higher Ca to diabetes risk [52]; conversely, Faysal et al. found lower Ca levels in diabetic individuals than in healthy controls [53].

In our cohort, albumin-corrected plasma Ca was numerically higher in T2DM with preserved renal function than in controls (89.0 vs. 86.7 mg/L), but this difference was not statistically significant, whereas the reduction in the reduced-eGFR group was significant both against controls (*p* < 0.05) and against T2DM with preserved eGFR (80.9 vs. 89.0 mg/L; *p* < 0.01). Within the T2DM cohort, therefore, albumin-corrected plasma Ca tracked renal function rather than being uniformly elevated or reduced in diabetes. Long-lasting hypercalciuria in diabetic patients and the inability to compensate by releasing Ca from bone may explain these changes [13]; the significantly lower Ca supply in the diabetic group and unmeasured urinary Ca leave increased calciuria unconfirmed [13].

The lower albumin-corrected plasma Ca concentration observed in patients with T2DM and reduced eGFR may reflect alterations in calcium homeostasis associated with impaired renal function. Mechanisms involving the FGF23–Klotho–vitamin D axis have been described in the context of kidney dysfunction; however, FGF23, α-Klotho, vitamin D metabolites, and parathyroid hormone were not measured in the present study. Therefore, the observed differences cannot be attributed to these mechanisms or interpreted as evidence of chronic kidney disease–mineral and bone disorder. Furthermore, differences in age and sex distribution between the study groups may have contributed to the observed associations.

Albumin-corrected Ca was used throughout; without correction, low plasma albumin in patients with T2DM and reduced eGFR would lower total Ca and confound the eGFR < 60 comparison [38,54]. Recent cross-sectional evidence confirms joint dysregulation of albumin-corrected Ca and 1,25(OH)_2_D_3_ in T2DM and diabetic kidney disease, with albumin-corrected Ca improving early detection of mineral disturbance [38].

Although albumin correction accounts for differences in plasma albumin concentrations, albumin-corrected total Ca does not necessarily reflect ionized Ca concentrations, particularly in patients with impaired renal function. Therefore, the lower albumin-corrected plasma Ca observed in the T2DM group with reduced eGFR should be interpreted as a difference in total calcium status rather than definitive evidence of hypocalcemia. The potential contribution of altered renal calcium handling and endocrine regulatory mechanisms remains uncertain, as ionized Ca, parathyroid hormone, FGF23, and vitamin D metabolites were not measured. These findings should not be interpreted as evidence of chronic kidney disease–mineral and bone disorder.

Blood tests alone cannot identify impaired Ca metabolism, as bone buffers plasma Ca [55]; other markers of Ca metabolism are needed. We found no significant increase in plasma Ca relative to controls and comparable whole-blood Ca; elevated Ca-related cardiovascular risk therefore cannot be confirmed in this cohort.

### 4.3. Iron

Plasma Fe concentrations were significantly higher in patients with T2DM and reduced eGFR than in controls, whereas no significant difference was observed in the T2DM group with preserved eGFR. Whole-blood Fe concentrations were significantly higher in both T2DM groups compared with controls. These findings are not entirely consistent with previous reports, including the study by Forte et al., which found no significant differences in median blood Fe concentrations between diabetic and non-diabetic individuals [56]. Differences in study populations, analytical matrices, and clinical characteristics may contribute to these discrepancies.

Following hematocrit-based recalculation, blood cell Fe concentrations were numerically higher in both T2DM groups than in controls; however, neither difference was statistically significant. This distinction is important because whole-blood Fe concentrations are strongly influenced by erythrocyte mass and hemoglobin-associated iron. Consequently, elevated whole-blood Fe should not be considered direct evidence of increased intracellular Fe accumulation or altered iron storage.

The hepcidin–ferroportin axis provides a plausible biological framework for disturbances in systemic iron homeostasis associated with inflammation and T2DM [9,10,11]. Nevertheless, our findings do not provide direct evidence of hepcidin-mediated intracellular iron sequestration. Hepcidin, ferritin, transferrin saturation, and inflammatory markers were not measured, and no significant between-group differences in calculated blood cell Fe concentrations were demonstrated. Therefore, the involvement of this pathway remains speculative and cannot be inferred from the present data alone.

An additional exploratory analysis examined the associations between calculated blood cell Fe concentrations and hematological indices in 45 participants with available matched data. Although correlations with MCHC were nominally significant in the two T2DM subgroups and exhibited opposite directions, neither remained significant after correction for multiple comparisons. Furthermore, calculated blood cell Fe and MCHC share a mathematical dependence on hematocrit, limiting the interpretation of these associations as independent biological relationships. The small subgroup sizes and absence of comprehensive iron-status measurements further restrict their clinical interpretation.The claim that erythrocyte Fe adds information beyond standard plasma indices must remain provisional. Hypochromic red cells and reticulocyte hemoglobin content are now available on routine analyzers as functional Fe markers [57], and automated ferritin immunoassays offer superior precision and faster throughput than manual ELISA [58]. Erythrocyte Fe may capture integrated body-iron status in a manner not captured by an acute-phase reactant such as ferritin, whereas ferritin remains the clinical standard for storage assessment [59]; other researchers have typically assessed Fe metabolism through indicators such as ferritin and transferrin [59,60]. Because ferritin and transferrin saturation were not measured here, we performed no head-to-head comparison of erythrocyte Fe against these standards; whether erythrocyte Fe provides complementary—rather than redundant—information is hypothesis-generating and must be tested in studies measuring both indices simultaneously. The predominantly female composition of the reduced-GFR group (22 of 30) is additionally relevant in this context, as iron stores differ by sex and menopausal status [61]; without such stratification, part of the between-group erythrocyte Fe difference could reflect these known sex differences rather than diabetes- or GFR-related pathophysiology. This limitation is particularly relevant when interpreting whole-blood Fe concentrations, given their dependence on erythrocyte mass and hemoglobin concentration.

### 4.4. Anthropometric and Biochemical Parameters

Elevated BMI was significantly associated with T2DM in both groups versus controls, in line with other studies [62]; glucose and HbA1c were significantly elevated, consistent with the literature [63]. Uric acid, urea and creatinine were significantly increased in the eGFR < 60 group; mild hyperuricemia has been intricately linked to CKD risk in T2DM [64], and elevated uric acid independently predicts T2DM onset in subjects with CKD [65]. ALT and AST were significantly elevated in the diabetic group with preserved renal function. Patients with decreased eGFR and T2DM had significantly higher whole-blood K than those with normal renal function; plasma K has been linked to declining eGFR in late CKD, with T2DM as a possible contributing risk factor [66], although we did not observe a significant T2DM–K association. In contrast to reports identifying lower creatinine as a predictor of T2DM [67], creatinine in the T2DM group with preserved eGFR did not differ significantly from controls.

### 4.5. Dietary Intake of Sodium, Potassium, Calcium, Magnesium, and Iron

T2DM patients, regardless of eGFR, consumed significantly less Na and Ca but more K and Mg than controls. Because the 3-day records were tabulated only at the aggregated T2DM level (Section 3.5) and were completed during hospitalization, this pattern cannot be attributed to either renal stratum or to habitual home intake, and no inferential comparison between the two GFR-based T2DM groups is presented. Whether the observed mineral differences are independent of dietary intake therefore cannot be determined from the present data. Dietary Fe did not differ significantly (*p* > 0.05).

A meta-analysis suggests that increasing Ca intake toward 750 mg/day may protect against diabetes development [68]; the control group’s average intake (730.72 mg/day) approaches this level. Several factors affect the final blood concentration of a given mineral elements: absorption and metabolism disorders, drugs used directly or indirectly (e.g., by affecting the intestinal microbiota [69]), and even physical activity, which can influence metabolic processes and, consequently, the bioavailability of mineral elements such as Fe [70].

### 4.6. Limitations

Renal function was assessed using eGFR at a single time point, and urinary albumin excretion was not evaluated. Consequently, the chronicity of renal impairment could not be established, and a formal diagnosis of chronic kidney disease could not be confirmed. Participants with T2DM were therefore classified solely according to preserved eGFR (≥60 mL/min/1.73 m^2^) or reduced eGFR (<60 mL/min/1.73 m^2^). This distinction describes renal function at the time of assessment and should not be interpreted as a classification of chronic kidney disease severity. Future studies should include repeated measurements of renal function and urinary albumin-to-creatinine ratio to enable a more comprehensive assessment of kidney disease.

Erythrocyte Ca could not be quantified (due to protocol sensitivity), leaving the intracellular Ca pool unassessed. Ionized Mg and the Ca/Mg ratio were not determined; total Mg underestimates the deficit in T2DM [49], and the Ca/Mg ratio discriminates glycemic-control quality and CAD risk better than Ca or Mg alone [46,47]. Even albumin-corrected total Ca does not reliably predict ionized Ca in CKD when total CO_2_ or albumin is low [71]. Erythrocyte mineral concentrations were calculated indirectly from whole-blood and plasma assays and therefore combine the measurement error of two determinations; direct intracellular measurement was unavailable. Dietary data were not disaggregated by renal strata and were collected on a hospital-prescribed diet, limiting inferences about habitual dietary drivers.The primary comparisons were univariate. The groups differed in age, sex, BMI, glycemic control and renal function, and mineral concentrations may be influenced by these factors; multivariable adjustment (e.g., age, sex, or BMI-adjusted models) was not performed because of the modest sample size (n = 91) and the limited number of events per stratum. The reported differences should therefore be confirmed in larger, confounder-adjusted analyses. In particular, the female predominance of the reduced-GFR group (22 of 30 women) may have influenced the Fe findings, given known sex differences in iron stores related to menstrual losses and menopausal status [72].

The exploratory correlation analysis with MCHC was limited to 45 participants with available matched data, and the small subgroup sizes reduced the precision of the correlation estimates. Furthermore, the shared dependence of calculated blood cell Fe concentrations and MCHC on hematocrit may contribute to mathematical associations between these variables.Numerous pairwise comparisons were performed across elements, blood compartments, dietary parameters and clinical variables without formal multiplicity correction, increasing the risk of Type I error; the four mineral outcomes of primary interest (plasma Mg, albumin-corrected plasma Ca, whole-blood Fe and calculated blood cell Fe) are reported as the principal comparisons, while the remaining contrasts are secondary and should be interpreted cautiously.

No a priori power calculation was performed; post hoc power estimates derived from the same data used for significance testing are uninformative about the probability of a true effect and are therefore deliberately not reported.

Anthropometric, biochemical, hematological and dietary variables departed from normality and were therefore analyzed nonparametrically with the Mann–Whitney U test (Section 2.5); mineral concentrations, in contrast, were compared using Welch’s ANOVA followed by the Games–Howell post hoc test, which does not assume equal variances between groups. All variables are presented as means ± SD in accordance with the reporting format used throughout the manuscript; medians with interquartile ranges are available from the corresponding author on request. Finally, self-reported 3-day diaries, the absence of urinary mineral element data, and a single-region Polish cohort limit generalizability. Sodium status is a specific instance of this problem and warrants separate mention: because Na intake is a determinant of Ca and Mg balance rather than a neutral background [17,18], and because the salt content of a hospital-prescribed diet need not equal that of the participants’ habitual home diet, the significant between-group difference in recorded Na intake (2328.52 ± 434.51 mg/day in T2DM vs. 2948.01 ± 364.21 mg/day in controls; *p* < 0.01) leaves open the possibility that part of the observed difference in plasma Mg and Ca reflects a sodium-driven change in mineral balance between the home and hospital settings rather than a T2DM-related disturbance of mineral metabolism. This possibility could not be quantified: no 24-h urine collection was performed, so urinary Na, Ca and Mg excretion were not measured; the Na content of the hospital menu as prepared was not available; and habitual pre-admission Na intake was not recorded. The sodium contribution to the observed Ca and Mg differences therefore remains unresolved and should be addressed in future balance studies with controlled dietary salt.

Renal function was originally assessed using the MDRD equation. An exploratory sensitivity analysis using the 2021 CKD-EPI creatinine equation was subsequently performed for all 91 participants using available individual-level clinical records. However, the analytical methods and IDMS traceability of the historical creatinine measurements could not be verified. Consequently, the CKD-EPI-based estimates should be interpreted cautiously. Five participants crossed the eGFR threshold of 60 mL/min/1.73 m^2^ in this exploratory analysis, indicating that the choice of estimation equation may influence group allocation.

A separate limitation concerns the retrospective availability of data for the additional exploratory correlation analyses performed during manuscript revision. The original statistical analyses were conducted using the full cohort of 91 participants. However, although hematological and mineral measurements had originally been collected, complete matched individual-level records could be recovered from the archived documentation for only 45 participants (18 controls, 17 patients with T2DM and preserved eGFR, and 10 patients with T2DM and reduced eGFR). This limitation concerns the retrospective recovery of data and does not imply that the original analyses were performed on an incomplete cohort. Nevertheless, the recovered subset may not be representative of the original study population, introducing potential selection bias. The small subgroup sizes, particularly in patients with reduced eGFR, also limit statistical power and the precision of the correlation estimates. Accordingly, these additional correlation analyses should be interpreted as exploratory and hypothesis-generating.

## 5. Conclusions

The present study identified blood compartment-dependent differences in Mg, Ca, and Fe concentrations between patients with T2DM and controls, with T2DM participants classified according to preserved eGFR (≥60 mL/min/1.73 m^2^) or reduced eGFR (<60 mL/min/1.73 m^2^). Patients with T2DM and reduced eGFR exhibited significantly lower plasma Mg and albumin-corrected plasma Ca concentrations than controls. Albumin-corrected plasma Ca was also significantly lower in the reduced-eGFR group than in patients with T2DM and preserved eGFR (*p* < 0.01). These findings describe associations with renal function at the time of assessment and should not be interpreted as differences between established stages of chronic kidney disease.

Following hematocrit-based recalculation, no statistically significant between-group differences were detected in blood cell Fe or Mg concentrations. These findings emphasize that differences observed in plasma and whole blood do not necessarily correspond to alterations in the calculated blood cell fraction.

Exploratory correlations between calculated blood cell Fe concentrations and hematological indices did not remain statistically significant after correction for multiple comparisons. Accordingly, the present findings do not establish intracellular Fe accumulation, renal function-dependent erythrocyte iron alterations, or hepcidin-mediated Fe sequestration.

The results support further investigation of mineral distribution across blood compartments in T2DM but do not establish the diagnostic superiority of calculated blood cell mineral concentrations over established biochemical markers. Prospective studies incorporating direct intracellular measurements, comprehensive iron-status assessment, ionized Ca and Mg measurements, and adjustment for relevant clinical confounders are needed to clarify their potential clinical significance.

## Figures and Tables

**Table 1:** Magnesium, calcium and iron concentrations (mg/L) in plasma, whole blood, and blood cells of the control group and T2DM subjects, and the significance of differences in elemental content across sample types and renal function groups.

Mineral Elements		Control Group (n = 32)			T2DM, Preserved eGFR (≥60 mL/min/1.73 m^2^) (n = 29)			T2DM, Reduced eGFR (<60 mL/min/1.73 m^2^) (n = 30)		
		Plasma	Whole Blood	Blood Cells	Plasma	Whole Blood	Blood Cells	Plasma	Whole Blood	Blood Cells
Magnesium [mg/L]	Mean	21.6	35.0	58.0	20.2	38.0 *	58.1	19.5 **	33.7 ††	54.9
	SD	2.1	4.5	11.4	2.7	3.9	7.0	2.8	2.6	5.7
Calcium [mg/L]	Mean	86.7	58.1	ND	89.0	52.6	ND	80.9 ^††^	53.9	ND
	SD	11.3	14.4	ND	8.7	6.4	ND	4.8	10.8	ND
Iron [mg/L]	Mean	5.05	347.0	913.6	5.18	403.9 *	1003.4	7.75 *	408.4 *	992.9
	SD	2.90	103.2	230.9	4.91	45.3	112.1	4.78	71.6	73.0

eGFR—estimated glomerular filtration rate, ND—not determined (erythrocyte Ca could not be quantified with the present protocol; see Methods), SD—standard deviation, T2DM—type 2 diabetes mellitus. Values are expressed as mean ± standard deviation (SD). Between-group differences were assessed using Welch’s ANOVA followed by the Games–Howell post hoc test. * *p* < 0.05 and ** *p* < 0.01 compared with the control group; †† *p* < 0.01 compared with the T2DM group with eGFR ≥ 60 mL/min/1.73 m^2^. Plasma calcium concentrations were corrected for albumin. Blood cell Fe and Mg concentrations were calculated using individual hematocrit values.

**Table 2:** Elemental contents of the diets of T2DM and control subjects.

Element	Control (n = 32)		T2DM (n = 59)		T2DM, Preserved eGFR (≥60 mL/min/1.73 m^2^) (n = 29)		T2DM, Reduced eGFR (<60 mL/min/1.73 m^2^) (n = 30)		*p*
	Mean	SD	Mean	SD	Mean	SD	Mean	SD	
Na (mg/day)	2948.01	364.21	2328.52	434.51	ND *	ND *	ND *	ND *	<0.01
K (mg/day)	2358.83	535.98	3033.39	585.76	ND *	ND *	ND *	ND *	<0.01
Ca (mg/day)	730.72	431.22	438.28	225.43	ND *	ND *	ND *	ND *	<0.01
Mg (mg/day)	249.63	53.21	309.36	44.65	ND *	ND *	ND *	ND *	<0.01
Fe (mg/day)	11.76	6.10	13.24	7.20	ND *	ND *	ND *	ND *	>0.05

Ca—calcium, Fe—iron, K—potassium, Mg—magnesium, Na—sodium, ND—not determined at the disaggregated level, T2DM—type 2 diabetes. The *p*-value was assessed using the Mann–Whitney U test for the original (aggregated) T2DM vs. Control contrast. The bolded results indicate statistically significant differences (*p* < 0.05, *p* < 0.01). * The three-cohort disaggregation (Control vs. T2DM eGFR ≥ 60 vs. T2DM eGFR < 60) is the methodological expectation of the present study. The three-day food-diary records were, however, originally tabulated only for “Control” and “T2DM (all)” in the source dataset. Disaggregated means and standard deviations for the T2DM sub-cohorts are therefore not available without re-analysis of the individual intake records, which is identified as an outstanding limitation in Section 4.6.

## Data Availability

The dataset used and analyzed during the current study is available from the corresponding author on reasonable request. The original dataset used for the primary statistical analyses is no longer available in a form permitting full reanalysis; for the additional exploratory correlation analyses, matched individual-level records were recovered retrospectively from archived laboratory documentation for 45 of the 91 participants (Section 2.5).

## Supplementary Materials

The following supporting information can be downloaded at: https://www.mdpi.com/article/10.3390/jcm15218727/s1, Table S1: Parameters of AAS determinations of individual elements; Table S2: Determinability of selected metals by AAS method; Table S3: The mineralization procedure used; Table S4: Demographic and clinical characteristics of participants (n = 91); Table S5: Mean values of anthropometric measurements, selected laboratory measures, blood pressure and the hematological characteristics in the study groups of patients by gender; Table S6: Characteristics of selected biochemical parameters in the studied groups of patients; Table S7: Statistics of biometric parameters and hematological indices between control the group and two groups of patients with type 2 diabetes in relation to renal function; Table S8: Spearman’s rank correlations between calculated blood cell iron concentration and hematological indices in participants with available matched data; Figure S1: PCA biplot of selected parameters for the control group; Figure S2: PCA biplot of selected parameters for the test group established based on eGFR ≥ 60 mL/min/1.73 m^2^; Figure S3: PCA biplot of selected parameters for the test group established based on eGFR < 60 mL/min/1.73 m^2^; Figure S4: 3D PCA biplot of selected parameters for all of the test groups. a) plot containing all of the parameters rotated to visualize the distinct covariance of uric acid; b) simplified plot—parameters were simplified and are represented by the highest impact component in the group; c) cumulative variance plot; Figure S5: Comparison of magnesium content in plasma, whole blood, and blood cell samples of the control group and type 2 diabetic subjects; Figure S6: Comparison of calcium content in serum, whole blood, and erythrocytes of the control group and type 2 diabetic subjects; Figure S7: Comparison of iron content in plasma samples, whole blood, and blood cells of the tested groups of the control group and T2DM patients.

## Abbreviations

The following abbreviations are used in this manuscript:

ACR	Albumin-to-creatinine ratio
ALT	Alanine aminotransferase
AAS	Atomic absorption spectrometry
AST	Aspartate aminotransferase
BP	Blood pressure
BMI	Body mass index
Ca	Calcium
CAD	Coronary artery disease
CKD	Chronic kidney disease
CKD-EPI	Chronic Kidney Disease Epidemiology Collaboration
eGFR	Estimated glomerular filtration rate
Fe	Iron
FG	Fasting glucose
GGTP	Gamma-glutamyl transpeptidase
HbA1c	Glycated hemoglobin
HDL	High-density lipoprotein
IDMS	Isotope Dilution Mass Spectrometry
K	Potassium
LDL	Low-density lipoprotein
MDRD	Modification of Diet in Renal Disease
Mg	Magnesium
Na	Sodium
ND	Not determined
SD	Standard deviation
T2DM	Type 2 diabetes mellitus
TCH	Total cholesterol
TG	Triglycerides
WHR	Waist–hip ratio
