# Supplementary material for: Beyond Plasma: Blood Fraction-Specific Patterns of Magnesium, Calcium and Iron in Type 2 Diabetes Across Preserved and Reduced Estimated Glomerular Filtration Rate
Source: J Clin Med. 2026 Nov 9;15(21):8727. doi: 10.3390/jcm15218727 (PMC13764025; doi:10.3390/jcm15218727)
Supplement: Supplementary file 1 [file jcm-15-08727-s001.zip › jcm-4607236-supplementary.pdf]

Supplementary materials

Beyond plasma: blood fraction-specific patterns of magnesium, calcium and iron in type 2 diabetes across preserved and reduced estimated glomerular filtration rate

The principles of blood sampling and processing.

From each patient, 7.5ml of venous blood was collected into tubes coated with lithium heparin in a closed vacuum system (Monovette Li-Hep 7.5ml). The samples were then divided into two equal parts. One was preserved for further study (frozen at -80oC), and the other was centrifuged for 5 minutes at about 2000rcf within an hour of collection. The plasma was centrifuged within 1 hour of collection to avoid the release of minerals from the cell into the plasma by lysis. A mildly decelerating centrifuge program was used for this purpose. At least 2ml of plasma was then separated and transferred to a separate tube. The tube with 2 ml of plasma and at least 2 ml of whole blood were used for further analysis.

Pressure microwave digestion of 1 milliliter of whole blood and 1 milliliter of plasma in 5 milliliters of spectrally pure nitric acid (V) [Merck] using an Ertec Magnum II microwave mineralizer was then used to remove the matrix effect. Appropriate dilutions of the mineralizate were prepared depending on the mineral to be determined. A standard curve was prepared based on standard solutions. Merck AAS Certipur certified refferece materials were used for calibration. An atomic absorption spectroscope manufactured by GBC model 932 plus was used in the study. The wavelength range of the monochromator was from 185 to 900 nm with a resolution of 0.2 nm. The emission sources were two lamps, a multi-cathode for the determination of Fe and a single-element cavity cathode for the determination of Ca and Mg. In this case, the dual beam optical system had the ability to correct background and flame emissions through asymmetric modulation with a measurement interval of 0.01 nm. Detailed assay parameters are shown in Supplementary Table S1. Merck AAS Certipur certified reference materials were used for calibration.

The contents of the selected ion in whole blood and plasma samples were directly determined by atomic absorption spectroscopy. The intracellular content of the selected mineral was determined as the difference between its concentration in whole blood and its concentration in plasma, corrected for hematocrit value.

In this study, the ASA method was used for the determination of metal ions. Routine determinations of the content of metal ions in blood or plasma samples usually use colorimetric methods, which involve measuring the absorbance of complexes of metal ions with various ligands, e.g., for the determination of magnesium (Mg), the formation of a color complex of magnesium with sodium azide is used. Colorimetric methods used in routine determinations use complex compounds with high stability. If there is no proper metabolic equilibrium in the cell, e.g., in the case of acidosis, various compounds can be formed in the cell that can act as ligands, e.g., β-hydroxybutyric acid, acetoacetate and others. These compounds have strong complex-forming abilities and can form stronger complexes with the metal ions being labeled than with the ligands used for colorimetric determination. In the case of sodium azide, stronger complexes are formed with citrates and oxalates. In such cases, the results obtained may be inadequate to the true concentration of the analyte being determined, i.e., they may be underestimated. The probability of the formation of various compounds that are products of improper metabolism (glucose, fatty acids) in the cell is high in diabetes mellitus, in which acidosis can occur, even if it is not clinically manifested.

To check the validity of the determinations carried out and the reliability of the results obtained, calcium and magnesium determinations were carried out in accordance with the procedure described. Supplementary Table S2 shows the results of these determinations. The declared content of calcium (Ca) in the standard reference serum (Seronorm) is 139mg/L, magnesium - 41mg/L, and iron (Fe) 2.15mg/L, while 143±4.1mg/L, 42.6±3.4mg/L and 2.10±0.13mg/L of these elements were determined, respectively. As can be seen from the table, these values are within the range of 95% agreement, and even more so within the range of acceptability. The analysis carried out gives confidence in properly developed procedures and determinations.

The standard mineralization procedure recommended by the manufacturer of the Ertec Magnum II mineralizer was used. The relevant parameters are shown in Supplementary Table S3. One-step digestion was carried out in a closed system with one digestion reagent.

Table S1. Parameters of AAS determinations of individual elements.

Element	wavelength [nm]	gap width [nm]	flame type	concentration [µg/ml]	sensitivity [µg/ml]
Ca	422.7	0.5	air-acetylene (oxidative)	1-4	0.02
Fe	248.3	0.2	air-acetylene (oxidative)	2-9	0.05
Mg	285.2	0.5	air-acetylene (oxidative)	0.1-0.4	0

K	769.9	0.5	air-acetylene (oxidative)	1.1-4.4	0.02
Na	330.2	0.5	air-acetylene (oxidative)	100-380	2.1

Ca – calcium, Fe – iron, K – potassium, Mg – magnesium, Na – sodium.

Table S2. Determinability of selected metals by AAS method.

	Ca [mg/L]	Mg [mg/L]	Fe [mg/L]
Declared content in the reference serum	139	41	2,15
Determined content in the reference serum	143	42,6	2,10
Standard deviation	4.1	3.4	0.13
Range of 95% compliance	134-144	39.2-42.8	2.05-2.25
Acceptable range	131-147	37.9-44.1	1.95-2.35

Ca – calcium, Fe – iron, Mg – magnesium.

Table S3. The mineralization procedure used.

Sample type	Sample volume	Solvent	Time	Power	Pressure range
Blood	1.0 ml	5 ml HNO_3	7 min.	100%	42-45 at
Plasma	1.0 ml	5 ml HNO_3	7 min.	100%	42-45 at

Table S4. Demographic and clinical characteristics of participants (n=91).

**Demographic variables**	**n (%)**
Gender:	
female	55 (60)
male	36 (40)
Educational level:	
low	32 (35)
medium	49 (54)
high	10 (11)
Physical activity level:	
low	56 (62)
medium	27 (29)
high	8 (9)
Duration of diabetes*	6.69 (3.25; 10)
Familial diabetes	45 (49)
Comorbidities:	
HA	76 (84)
CHF	17 (19)
CAD	35 (38)
Treatment	
ACEI	65 (71)
ASA	53 (58)
Statins	52 (57)
Fenofibrat	1 (1)
BB	20 (22)
MET monotherapy	10 (11)
PSU monotherapy	13 (14)
Insulin monotherapy	9 (10)
MET with SU	21 (23)

MET with insulin	3 (3)
Acarbose	1 (1)

ACEI – angiotensin-converting enzyme inhibitor, ASA – acetylosalic acid, BB – beta blocker, CAD – coronary artery disease, CHF – chronic heart failure, HA – hypertension, MET – metformin, SU – sulfonylureas.
Data for Duration of diabetes are shown as a median (minimum; maximum).
** Accodring to actual KDIGO Clinical Practice Gudelines.

Table S5. Mean values of anthropometric measurements, selected laboratory measures, blood pressure and the hematological characteristics in the study groups of patients by gender.

Parameters	Control group (n=32)			T2DM, preserved eGFR (≥60 mL/min/1.73 m²) (n=29)			T2DM, reduced eGFR (<60 mL/min/1.73 m²) (n=30)		
	Overall	Women	Men	Overall	Women	Men	Overall	Women	Men
Age [years]	68.25±15.30	71.94±13.32	64.56±16.67	60.51±9.86	61.35±11.82	59.33±6.44	75.13±6.91	75.59±6.97	73.87±7.08
BMI [kg/m^2]	26.35±4.68	26.39±4.77	26.32±4.75	30.84±5.55	31.7±6.78	29.63±2.98	31.43±6.49	31.87±7.29	30.21±3.6
WHR	0.945±0.071	0.904±0.065	0.982±0.056	0.936±0.075	0.894±0.059	0.996±0.052	0.951±0.055	0.936±0.050	0.993±0.049
SBP [mmHg]	126.1±17,7	129.7±15.1	122.5±19.8	129.7±18.7	131.2±20.2	127.5±17.1	137.2±27.8	137.9±28.1	135.0±28.9
DBP [mmHg]	75.9±11.6	75.0±8.16	76.9±14.5	74.4±7.69	75.2±6.91	73.3±8.87	75.7±14.3	77.3±14.5	71.3±13.6
Sodium [mmol/l]	140.6±5.8	141.5±7.4	139.8±3.6	133.9±23.2	131.1±30.3	137.8±2.1	139.7±3.84	140.6±3.55	137.1±3.60
Potassium [mmol/l]	4.26±0.43	4.16±0.48	4.35±0.37	4.15±0.33	4.18±0.31	4.11±0.37	4.42±0.50	4.36±0.54	4.61±0.32
WBC [10^3/µl]	7.74±3.12	7.89±3.85	7.59±2.29	7.52±2.25	7.43±2.38	7.65±2.16	8.12±2.31	8.08±2.14	8.24±2.91
RBC [10^6/µl]	4.32±0.76	4.20±0.71	4.44±0.83	4.52±0.49	4.46±0.49	4.61±0.49	4.40±0.84	4.35±0.73	4.48±1.14
Hb [g/dl]	12.75±2.38	12.28±2.12	13.22±2.60	13.31±1.58	13.18±1.49	13.49±1.77	12.67±2.29	12.54±2.22	13.03±2.60
HCT [%]	38.28±6.41	37.2±5.43	39.36±7.31	39.97±4.04	39.48±4.43	40.66±3.47	38.24±6.57	37.80±5.90	39.44±8.49

MCV [fl]	89.21±9.86	89.75±11.81	88.67±7.81	88.56±4.62	88.58±3.20	88.54±6.28	87.66±4.49	87.26±4.11	88.78±5.56
MCH [pg]	31.40±11.97	33.4±16.4	29.39±4.16	30.10±2.26	29.97±1.78	30.29±2.88	29.35±2.16	29.32±2.17	29.43±2.27
MCHC [g/dl]	33.22±1.75	32.93±1.71	33,51±1.76	33.97±1.34	33.84±1.43	34.15±1.24	33.11±0.98	33.10±0.98	33.14±0.98
PLT [10^3/μl]	231.1±63.5	241.9±61.6	220.2±65.6	230.4±68.7	226.8±68.2	235.5±79.6	237.0±101.6	256.4±109.9	185.0±48.6

BMI – body mass index, DBP – diastolic blood pressure, GFR – glomerular filtration rate, Hb – hemoglobin, HCT – hematocrit, MCH – mean corpuscular hemoglobin, MCHC – mean corpuscular hemoglobin concentration, MCV – mean corpuscular value, PLT – platelets. RBC – red blood cells, SBP – Systolic blood pressure, T2DM – type 2 diabetes mellitus, WHR – waist-hip ratio.
Data are expressed as mean value ± standard deviation.

Table S6. Characteristics of selected biochemical parameters in the studied groups of patients.

Parameters	Control group (n=32)			T2DM, preserved eGFR (≥ 60 mL/min/1.73 m²) (n=29)			T2DM, reduced eGFR (< 60 mL/min/1.73 m²) (n=30)		
	Overall	Women	Men	Overall	Women	Men	Overall	Women	Men
Glucose [mmol/l]	5.65±1.89	5.96±2.38	5.35±1.24	9.84±4.94	10.14±5.32	9.42±4.54	9.16±4.25	8.78±4.20	10.23±4.49
HbA1c [%]	5.75±0.46	5.73±0.52	5.76±0.41	8.78±2.42	8.83±2.50	8.71±2.40	7.98±2.39	7.51±1,84	9.24±3.30
Uric acid [μmol/l]	312.8±115.9	318.8±141.7	306.9±87.1	294.5±89.1	279.8±59.7	314.1±118.0	382.4±143.1	371.9±145.8	411,3±140.6
Urea [mmol/l]	5.90±1.74	5.98±2.03	5.82±1.46	5.22±1.17	5.10±1.26	5.38±1.04	10.78±4.26	10.38±4.37	11.87±4.01
Creatinine [μmol/l]	68.56±12.0	65.31±12.0	71.81±11.5	63.14±13.8	59.18±10.4	68.8±16.3	136.5±65.1	128.5±69.3	158.4±48.9
TCH [mmol/l]	4.32±1.27	4.5±1.39	4.14±1.15	4.75±2.20	4.97±2.79	4.45±0.94	4.66±1.09	4.66±1.05	4.64±1.29
LDL [mmol/l]	2.67±1.21	2.84±1.30	2.50±1.14	2.61±0.96	2.46±0.89	2.83±1.04	2.89±1.08	2.88±1.06	2.92±1.20
HDL [mmol/l]	1.17±0.46	1.20±0.45	1.13±0.48	1.15±0.31	1.25±0.30	1.02±0.28	1.11±0.35	1.14±0.32	1.03±0.42
TG [mmol/l]	1.39±0.52	1.34±0.38	1.45±0.65	2.02±1.45	2.07±1.83	1.94±0.73	1.80±1.00	1.69±0.70	2.10±1.58

ALT [U/I]	25.8±15.9	17.4±8.3	33.9±17.5	35.7±51.5	24.8±23.9	51.6±74.0	22.7±13.3	20.3±8.5	29.3±21.2
AST [U/I]	29.6±28.5	24.2±16.4	35.1±36.7	28.2±22.2	23.5±17.7	34.8±26.8	26.9±17.1	23.5±9.7	36.5±28.0
Bilirubin [μmol/l	10.92±5.03	9.94±3.44	11.89±6.20	10.59±6.67	9.46±6.07	12,20±7.41	10,57±5.57	9.50±4.65	13.51±7.07
GGTP [U/I]	29.72±24.17	20.19±9.89	39.26±30.25	66.90±96.63	36.47±28.71	110.0±138.3	39.80±34.81	30.59±17.12	65.13±56.05

ALT - Alanine aminotransferase, AST - Aspartate transaminase, GGTP – gamma-glutamyl transpeptidase, GFR – glomerular filtration rate, HbA1c – glycated hemoglobin, HDL – high-density lipoproteins, LDL – low-density lipoproteins, T2DM – type 2 diabetes mellitus, TCH – total cholesterol TG - triglycerides.
Data are expressed as mean value ± standard deviation.

Table S7. Statistics of biometric parameters and hematological indices between control the group and two groups of patients with type 2 diabetes in relation to renal function.

Parameter	T2DM, preserved eGFR (≥ 60 mL/min/1.73 m²) (n=29) vs control	T2DM, reduced GFR (< 60 mL/min/1.73 m²) (n=30) vs control	T2DM groups vs each other
BMI [kg/m^2]	**p<0.01**	**p<0.01**	p>0.05
WHR	p>0.05	p>0.05	p>0.05
SBP [mmHg]	p>0.05	p>0.05	p>0.05
DBP [mmHg]	p>0.05	p>0.05	p>0.05
Sodium [mmol/l]	p>0.05	p>0.05	p>0.05
Potassium [mmol/l]	p>0.05	p>0.05	p<0.01
WBC [10^3/μl]	p>0.05	p>0.05	p>0.05
RBC [10^6/μl]	p>0.05	p>0.05	p>0.05
Hb [g/dl]	p>0.05	p>0.05	p>0.05
HCT [%]	p>0.05	p>0.05	p>0.05
MCV [fl]	p>0.05	p>0.05	p>0.05
MCH [pg]	p>0.05	p>0.05	p>0.05
MCHC [g/dl]	p>0.05	p>0.05	p>0.05
PLT [10^3/μl]	p>0.05	p>0.05	p>0.05
Glucose [mmol/l]	**p<0.01**	**p<0.01**	p>0.05
HbA1c [%]	**p<0.01**	**p<0.01**	p>0.05
Uric acid [μmol/l]	p>0.05	**p<0.05**	**p<0.05**
Urea [mmol/l]	p>0.05	**p<0.01**	**p<0.01**
Creatinine [μmol/l]	p>0.05	**p<0.01**	**p<0.01**
TCH [mmol/l]	p>0.05	p>0.05	p>0.05
LDL [mmol/l]	p>0.05	p>0.05	p>0.05
HDL [mmol/l]	p>0.05	p>0.05	p>0.05
TG [mmol/l]	p>0.05	p>0.05	p>0.05
ALT [U/I]	**p<0.05**	p>0.05	p>0.05
AST [U/I]	**p<0.05**	p>0.05	p>0.05
Bilirubin [μmol/l]	p>0.05	p>0.05	p>0.05
GGTP [U/I]	p>0.05	p>0.05	p>0.05

ALT - Alanine aminotransferase, AST - Aspartate transaminase, BMI – body mass index, DBP – diastolic blood pressure, GGTP – gamma-glutamyl transpeptidase, GFR – glomerular filtration rate, Hb – hemoglobin, HCT – hematocrit, HDL – high-density lipoproteins, HbA1c – glycated hemoglobin, LDL – low-density lipoproteins, MCH - mean corpuscular hemoglobin, MCHC – mean corpuscular hemoglobin concentration, MCV – mean corpuscular value, PLT – platelets, RBC – red blood

cells, SBP – systolic blood pressure, T2DM – type 2 diabetes mellitus, TCH – total cholesterol, TG – triglycerides, WBC – white blood cells, WHR – waist-hip ratio.
p-value assessed using the Mann–Whitney U test.
The bolded results indicate statistically significant differences.

Table S8. Spearman's rank correlations between calculated blood cell iron concentration and hematological indices in participants with available matched data

Group	Hematological parameter	Spearman's r_s	Raw p-value	FDR-adjusted p-value
**Control group (n=18)**	MCV	0.290	0.243	0.437
	MCH	0.063	0.804	0.852
	MCHC	0.114	0.653	0.852
**T2DM, preserved eGFR (≥60 mL/min/1.73 m²) (n=17)**	MCV	0.049	0.852	0.852
	MCH	0.361	0.155	0.437
	MCHC	0.584	0.0138	0.0711
**T2DM, reduced eGFR (<60 mL/min/1.73 m²) (n=10)**	MCV	−0.091	0.803	0.852
	MCH	−0.442	0.200	0.437
	MCHC	−0.733	0.0158	0.0711

MCV - mean corpuscular volume, MCH - mean corpuscular hemoglobin, MCHC - mean corpuscular hemoglobin concentration, T2DM - type 2 diabetes mellitus, GFR - estimated glomerular filtration rate.
Data are presented as Spearman's rank correlation coefficients (r_s), with corresponding two-sided raw and false discovery rate (FDR)-adjusted p-values. The Benjamini–Hochberg procedure was applied to nine within-group comparisons (three hematological parameters × three study groups). Nominally significant correlations (raw $p < 0.05$) are indicated in bold. None of the associations remained statistically significant after FDR correction (adjusted $p > 0.05$). The analysis was restricted to 45 participants for whom matched individual-level data were available. An additional limitation concerns the availability of individual-level data for the exploratory correlation analyses. Although hematological and mineral measurements were originally collected for the study participants, complete matched records could be recovered from the archived documentation for only 45 of the 91 individuals (18 controls, 17 patients with T2DM and preserved eGFR, and 10 patients with T2DM and reduced eGFR). The incomplete recovery of historical records may have introduced selection bias, and the analyzed subset cannot be assumed to be representative of the full cohort. In addition, the small subgroup sizes, particularly in patients with reduced eGFR, limited statistical power and the precision of correlation estimates. These analyses should therefore be interpreted as exploratory and hypothesis-generating.

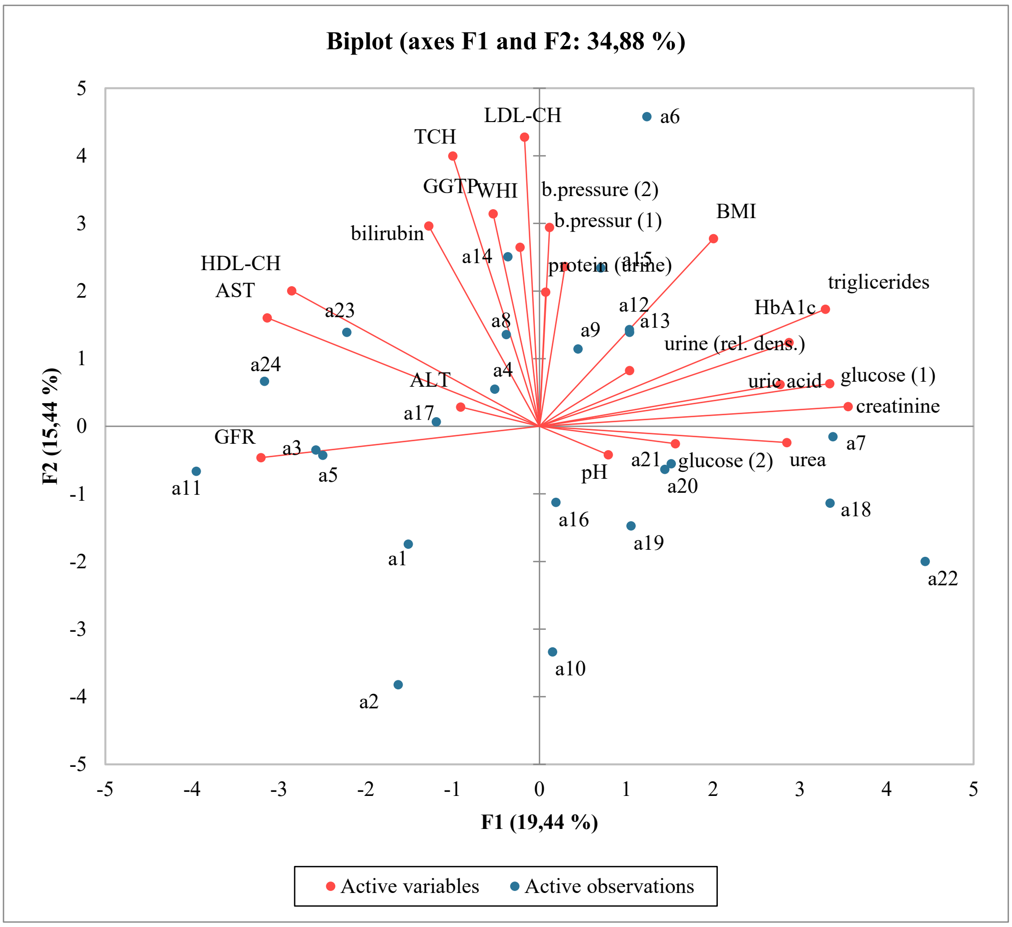

Figure S1. PCA biplot of selected parameters for the control group.

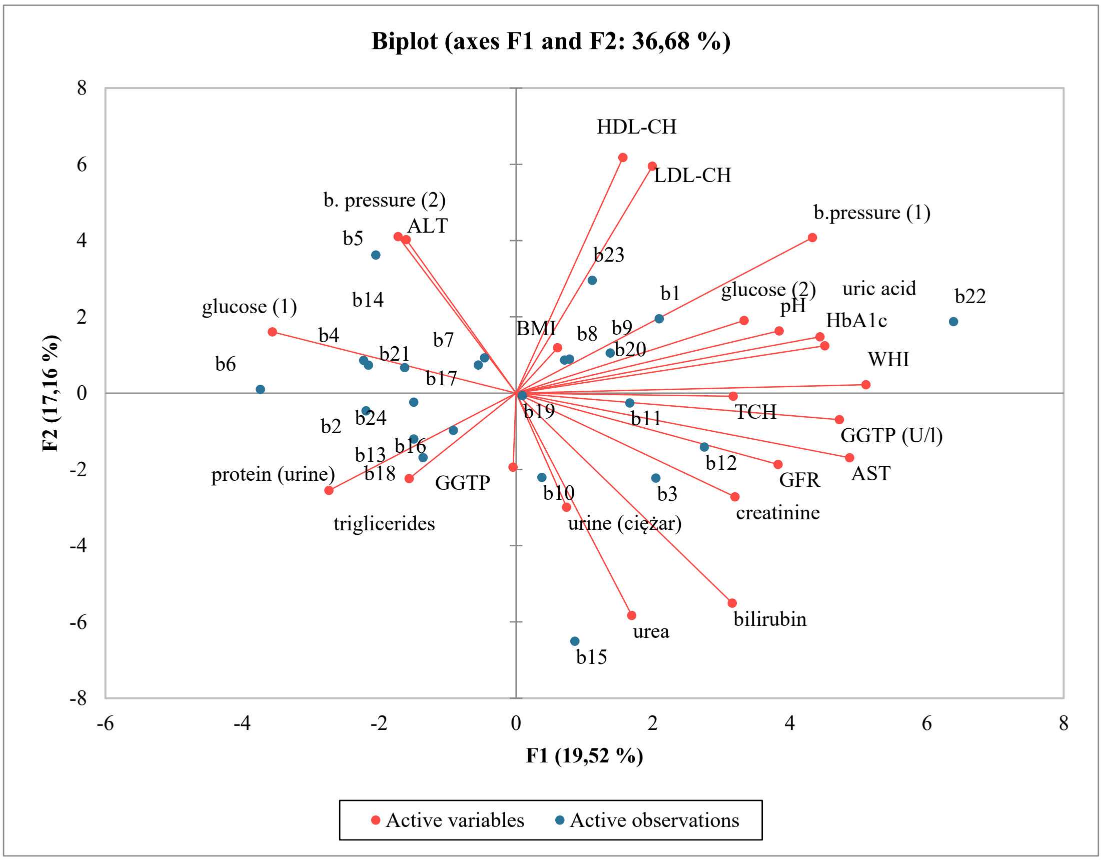

Figure S2. PCA biplot of selected parameters for the test group established based on eGFR ≥ 60 ml/min/1,73 m2.

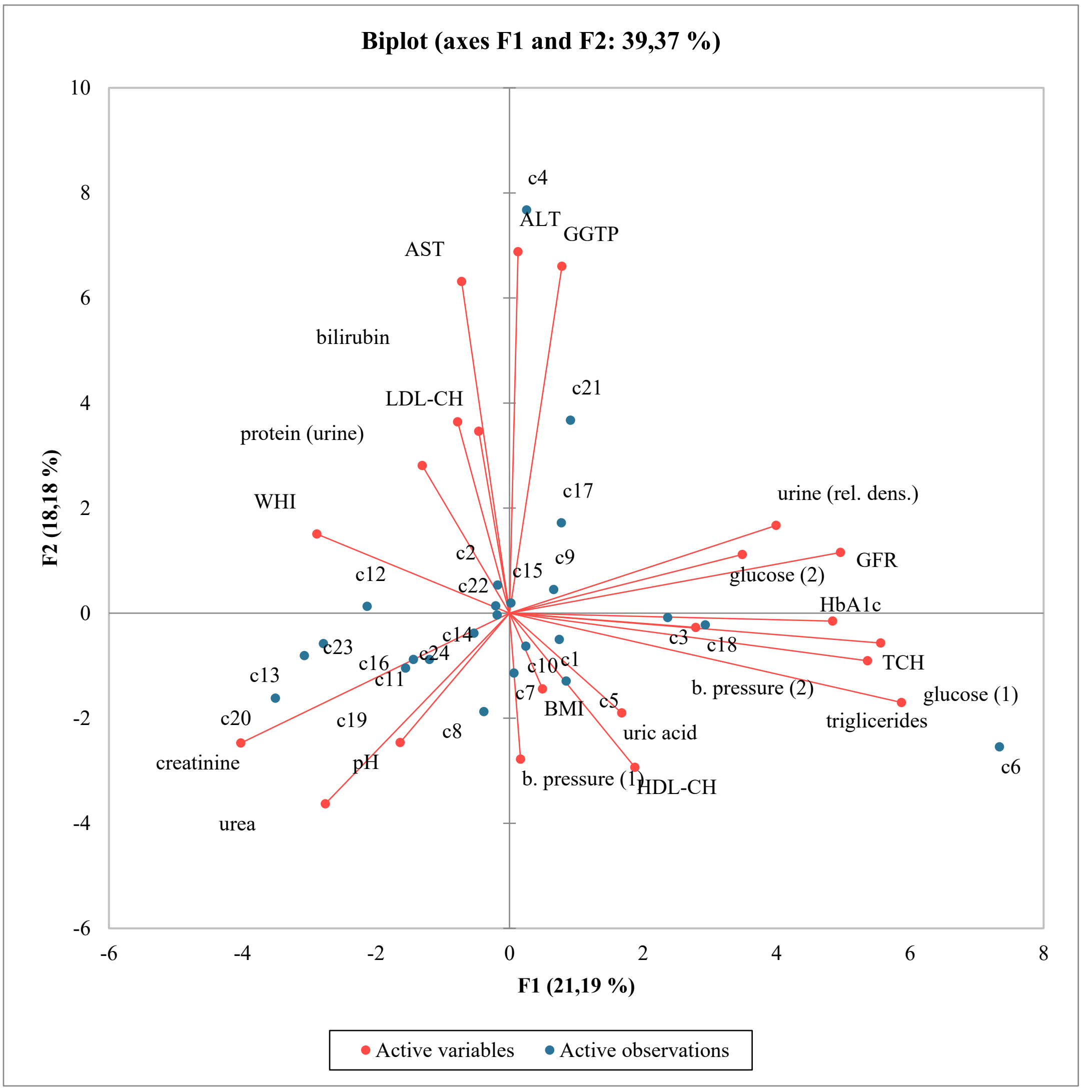

Figure S3. PCA biplot of selected parameters for the test group established based on eGFR < 60 ml/min/1.73 m2.

Figure S4. PCA of clinical and biochemical variables (n = 72)

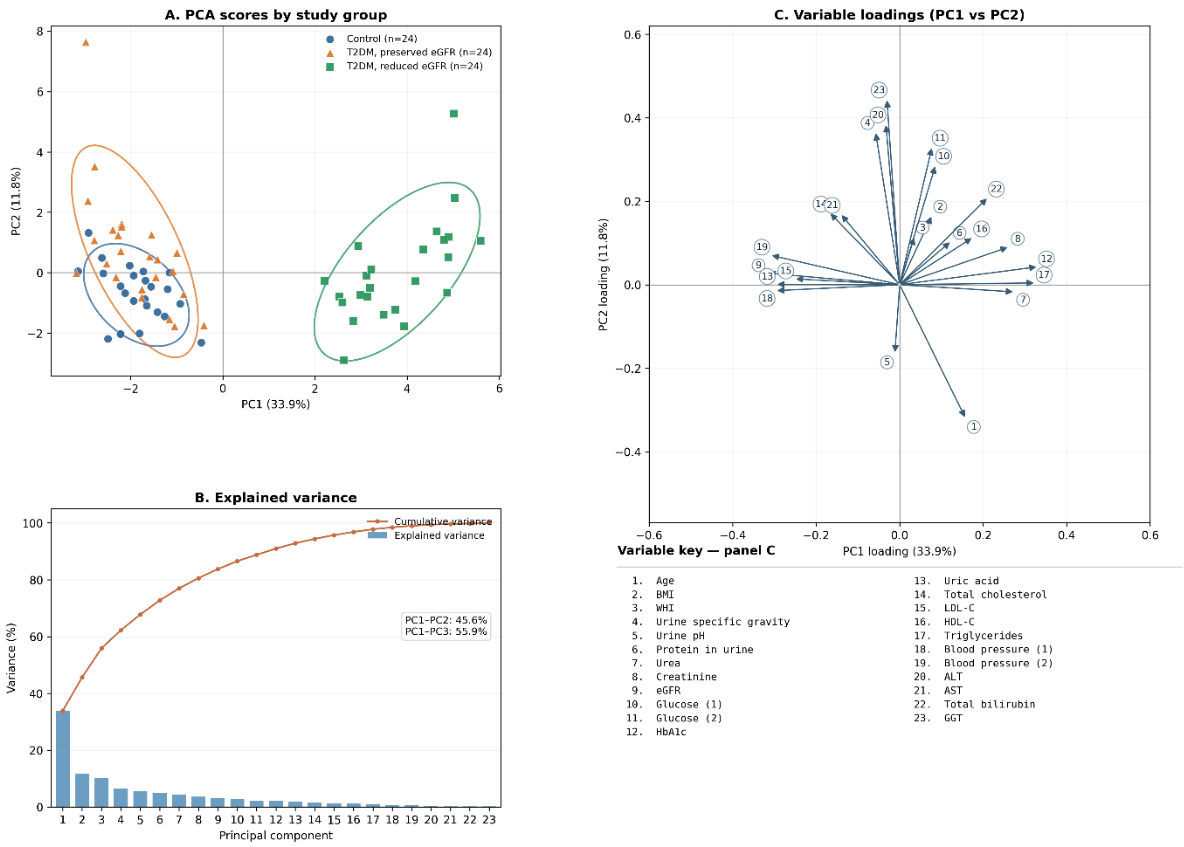

Figure S4. Principal component analysis of clinical and biochemical variables in the study groups.

(A) Score plot of PC1 versus PC2 for all participants (n = 72), with symbols indicating the control group, T2DM with preserved eGFR, and T2DM with reduced eGFR.

(B) Scree plot showing the percentage of explained variance for each principal component and cumulative explained variance.

(C) Loading plot of PC1 versus PC2. Variables are numbered for readability; their identities are provided in the legend within the panel.

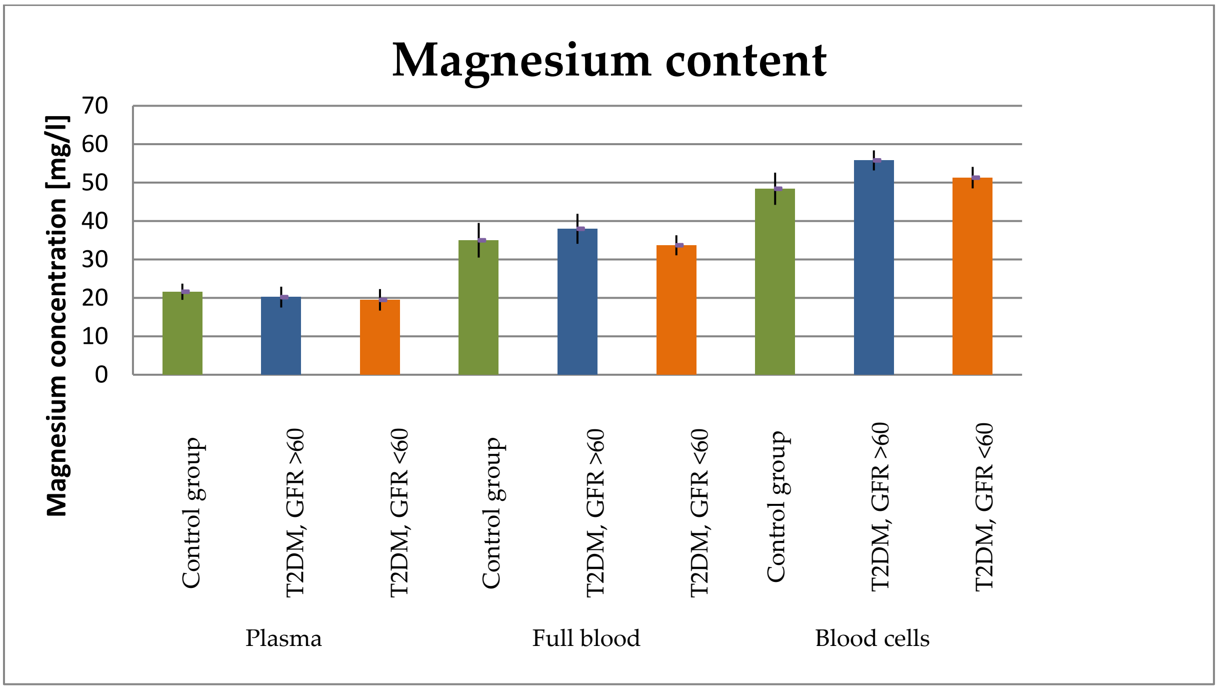

GFR – glomerular filtration rate, T2DM – type 2 diabetes mellitus.

Figure S5. Comparison of magnesium content in plasma, whole blood, and blood cell samples of the control group and type 2 diabetic subjects.

GFR – glomerular filtration rate, T2DM – type 2 diabetes mellitus.

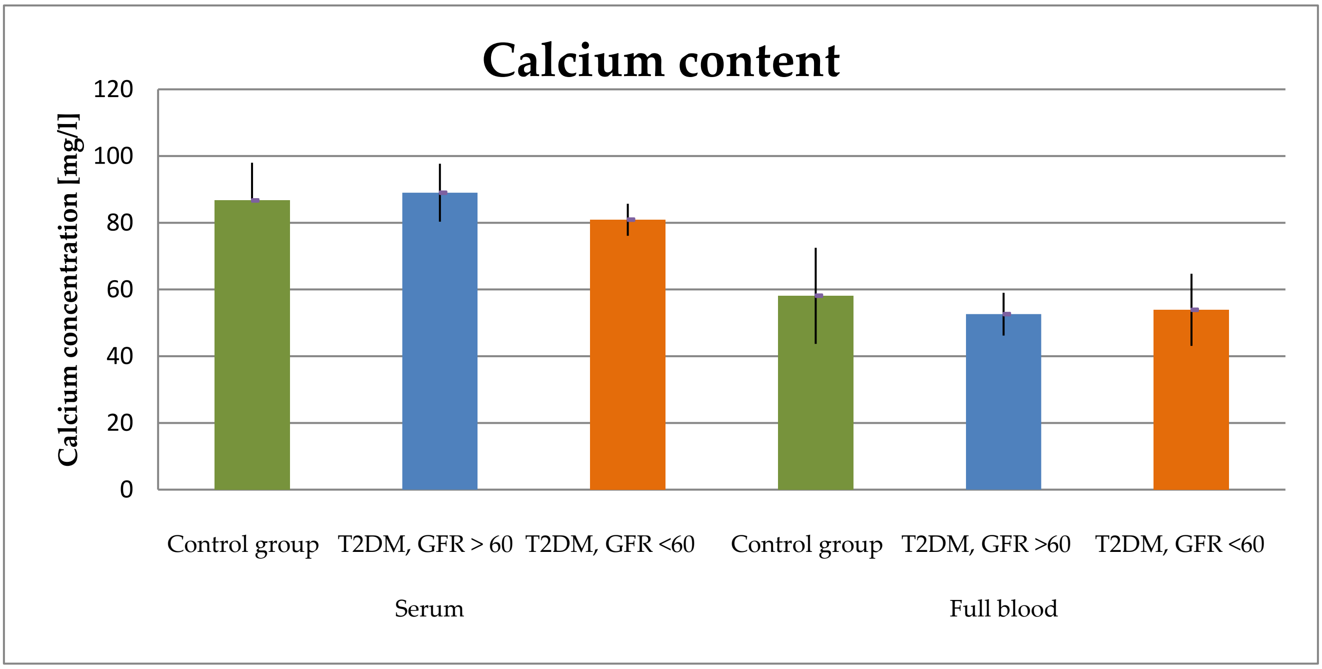

Figure S6. Comparison of calcium content in serum, whole blood, and erythrocytes of the control group and type 2 diabetic subjects.

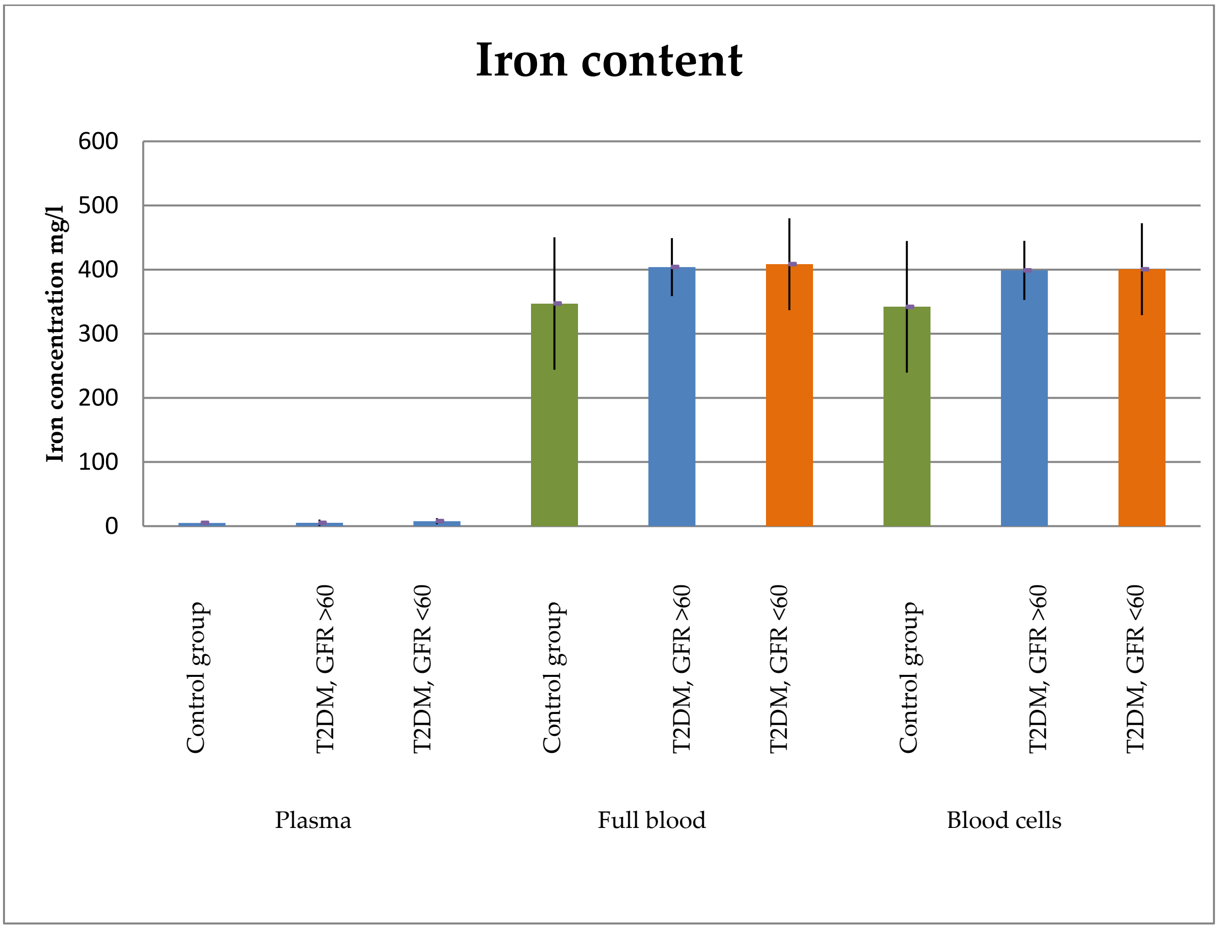

GFR – glomerular filtration rate, T2DM – type 2 diabetes mellitus.

Figure S7. Comparison of iron content in plasma, whole blood, and blood cells of the tested groups of the control group and T2DM patients.
